# Draft genome sequence of *Halopiger salifodinae* KCY07-B2^T^, an extremly halophilic archaeon isolated from a salt mine

**DOI:** 10.1186/s40793-015-0113-y

**Published:** 2015-12-10

**Authors:** Wei-Yan Zhang, Jing Hu, Jie Pan, Cong Sun, Min Wu, Xue-Wei Xu

**Affiliations:** College of Life Sciences, Zhejiang University, Hangzhou, 310058 P. R. China; Second Institute of Oceanography, State Oceanic Administration, Hangzhou, 310012 P. R. China

**Keywords:** *Halopiger salifodinae*, Archaea, Extreme halophile, Genome, Salt mine

## Abstract

*Halopiger salifodinae* strain KCY07-B2^T^, isolated from a salt mine in Kuche county, Xinjiang province, China, belongs to the family *Halobacteriaceae*. It is a strictly aerobic, pleomorphic, rod-shaped, Gram-negative and extremely halophilic archaeon. In this work, we report the features of the type strain KCY07-B2^T^, together with the draft genome sequence and annotation. The draft genome sequence is composed of 83 contigs for 4,350,718 bp with 65.41 % G + C content and contains 4204 protein-coding genes and 50 rRNA genes.

## Introduction

The genus *Halopiger*, which belongs to the family *Halobacteriaceae*, was originally established in 2007 by Gutiérrez et al. [1]. The type species of the genus *Halopiger* is *Halopiger xanaduensis* SH-6^T^. To date, the genus is comprised of three validly published species and two effectively but not validly published species: *H. xanaduensis* [1], *Halopiger aswanensis* [2], *Halopiger salifodinae* [3], *Halopiger djelfamassiliensis* [4] and *Halopiger goleamassiliensis* [5]. The species of the genus were reported to be isolated from hypersaline environments such as salt lake sediment [1, 4, 5], hypersaline soil [2] and salt mine [3]. All are Gram-negative, strictly aerobic and extremely halophilic [1–5]. In this genus, three genome sequences, including one finished genome sequence *H. xanaduensis* SH-6^T^, and two draft genome sequences *H. djelfamassiliensis* IIH2^T^ and *H. goleamassiliensis* IIH3^T^, are available in *Standards in Genomic Sciences* [4–6], except *H. aswanensis* 56^T^ which showed highest 16S rRNA gene similarity to *H. xanaduensis* SH-6^T^ (99.1 %). Here we present a summary of the classification and a set of features of strain *H. salifodinae* KCY07-B2^T^, together with a description of the non-contiguous finished genomic sequencing and annotation.

## Organism Information

### Classification and features

**Fig. 1:**
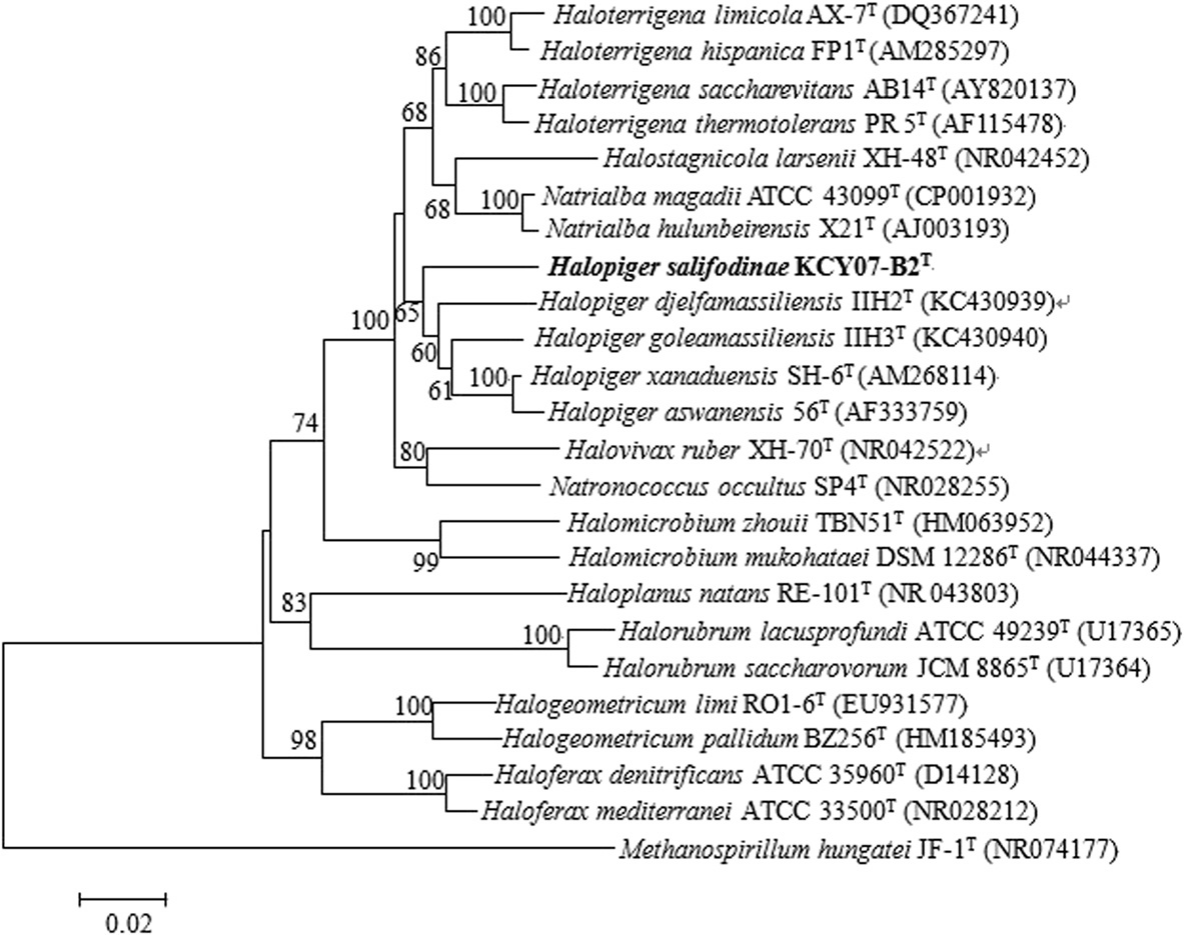
Neighbor-joining phylogenetic tree based on 16S rRNA gene sequences showed the relationship of *H. salifodinae* KCY07-B2^T^ and other related haloarchaeal species. GenBank accession numbers were indicated in parentheses. Bootstrap values based on 1000 replicates were shown for branches with more than 60 % support. Bar, 0.02 substitutions per nucleotide positions. *Methanospirillum hungatei* JF-1^T^ [30] was used as outgroup

A representative genomic 16S rRNA gene sequence of *H. salifodinae* KCY07-B2^T^ was compared with sequences deposited in the GenBank database using BLASTN [7]. The 16S rRNA gene sequence analysis showed that *H. salifodinae* KCY07-B2^T^ shared the highest sequence identities to *H. xanaduensis* SH-6^T^ (95.8 %), followed by *H. aswanensis* 56^T^ (95.5 %), *H. djelfamassiliensis* IIH2^T^ (94.9 %) and *H. goleamassiliensis* IIH3^T^ (94.8 %), and shared low sequence similarities (<94.8 %) to species of other genera. The phylogenetic tree was reconstructed by the neighbor-joining method using MEGA 5 and Kimura’s 2-parameter model for distance calculation [8, 9]. The phylogenetic tree was assessed by boot-strapping for 1000 replications, and the consensus tree was shown in Fig. 1.

**Fig. 2:**
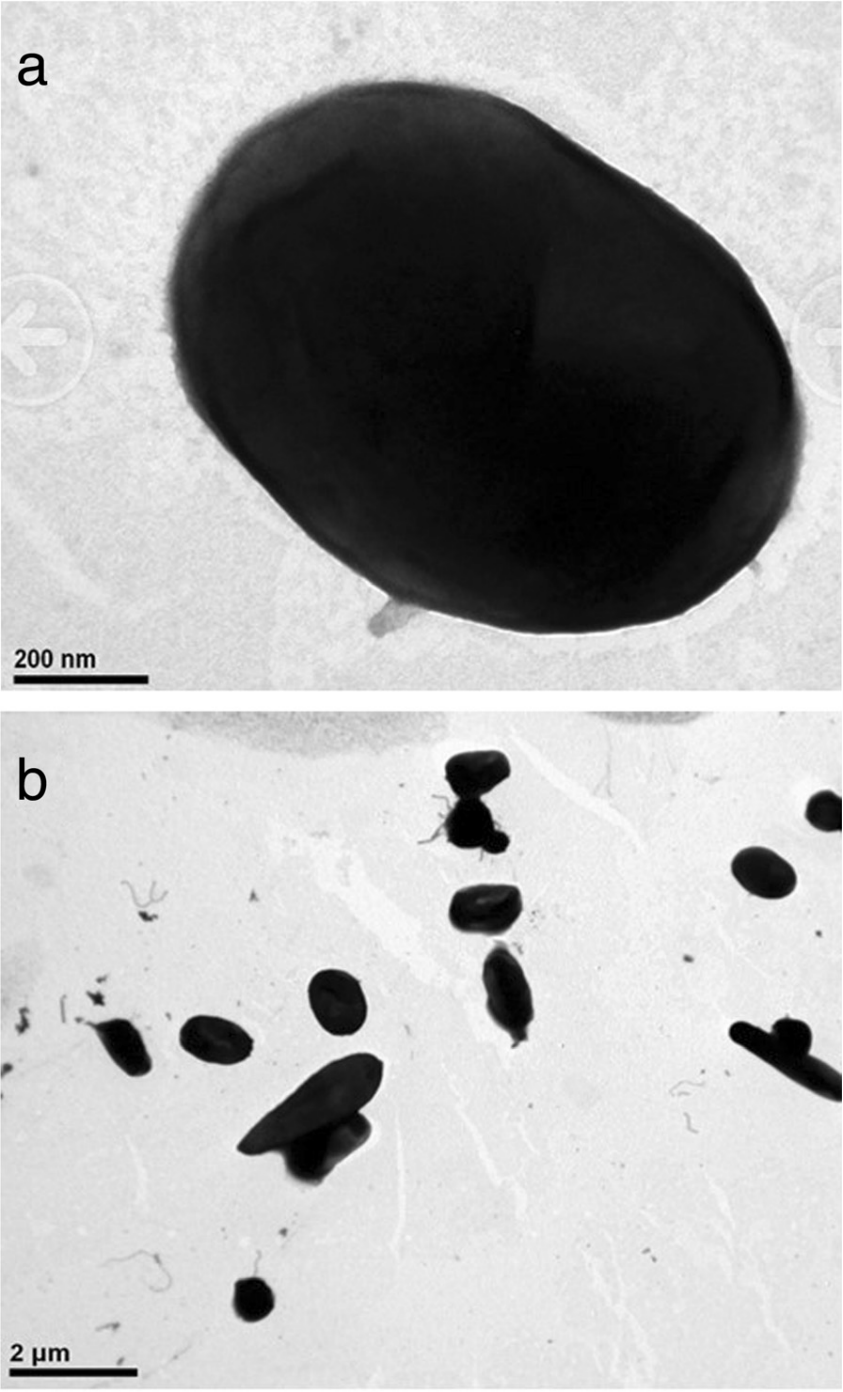
Electron micrographs of cells of strain KCY07-B2^T^ grown in liquid medium under optimum condition. **a** Transmission electron micrographs of strain KCY07-B2^T^ revealing rod-shaped, bar, 0.2 μm, **b** Showing a mixture of pleomorphic cells including short and long rod-shaped, bar, 2 μm

*H. salifodinae* KCY07-B2^T^ can tolerant high salinity (5.4 M NaCl ) and high temperature (50 °C) [3]. Cells lyse in distilled water. The optimal growth condition of strain KCY07-B2^T^ occured in medium NOM-3 with 2.9–3.4 M NaCl [3]. The optimum temperature was 37–45 °C. The optimum pH was 7.0, with a growth range of pH 6.0–8.0 [3]. Cells of strain KCY07-B2^T^ are strictly aerobic, non-motile and pleomorphic rod-shaped (Fig. 2). Several sugars, organic acids and amino acids can serve as sole carbon and energy sources, and amino acids are not required in the growth medium [3]. The features of *H. salifodinae* KCY07-B2^T^ are listed in Table 1.

**Table 1:** Classification and general features of *H. salifodinae* KCY07-B2^T^ according to the MIGS recommendations [10]

MIGS ID	Property	Term	Evidence code^a^
	Current classification	Domain *Archaea*	TAS [31]
		Phylum *Euryarchaeota*	TAS [32]
		Class *Halobacteria*	TAS [33, 34]
		Order *Halobacteriales*	TAS [35–37]
		Family *Halobacteriaceae*	TAS [38, 39]
		Genus *Halopiger*	TAS [1]
		Species *Halopiger salifodinae*	TAS [3]
		Type strain: strain KCY07-B2^T^ = JCM 18547^T^ = CGMCC 1.12284^T^	TAS [3]
	Gram stain	negative	TAS [3]
	Cell shape	pleomorphic rods	TAS [3]
	Motility	non-motile	TAS [3]
	Sporulation	non-sporulating	NAS
	Temperature range	25–50 °C	TAS [3]
	Optimum temperature	37–45 °C	TAS [3]
	pH range; Optimum	6.0–8.0; 7.0	TAS [3]
	Carbon source	acetate, _L_-asparagine, citrate, fumarate, _D_-glucose, _L_-glutamate, glycine, isoleucine, _L_-lysine, _L_-malate, _D_-mannose, _L_-serine_, D_-sorbitol, starch, succinate and _L_-threonine	TAS [3]
	Energy metabolish	heterotrophic	IDA
MIGS-6	Habitat	salt mine	TAS [3]
MIGS-6.3	Salinity	1.9–5.4 M NaCl (optimum 2.9–3.4 M)	TAS [3]
MIGS-22	Oxygen requirement	aerobic	TAS [3]
MIGS-15	Biotic relationship	free-living	IDA
MIGS-14	Pathogenicity	non-pathogenic	NAS
	Biosafety	1	NAS
MIGS-4	Geographic location	Kuche county, Akesu area in Xinjiang province, P.R. China	TAS [3]
MIGS-5	Sample collection time	2009	IDA
MIGS-4.1	Latitude	not reported	
MIGS-4.2	Longitude	not reported	
MIGS-4.4	Altitude	not reported	

^a^Evidence codes, *IDA* Inferred from Direct Assay, *TAS* Traceable Author Statement (i.e., a direct report exists in the literature), *NAS* Non-traceable Author Statement (i.e., not directly observed for the living, isolated sample, but based on a generally accepted property for the species, or anecdotal evidence). These evidence codes are from the Gene Ontology project [40, 41]

## Genome sequencing information

### Genome project history

This genome was selected for sequencing on the basis of its phylogenetic position and 16S rRNA sequence similarity to other members of the genus *Halopiger*. This whole genome shotgun project of strain *H. salifodinae* KCY07-B2^T^ was deposited at DDBJ/EMBL/GenBank under the accession number JROF00000000 and the sequence consisted of 83 contigs (further assembling constructed these contigs into 81 scaffolds). Table 2 shows the project information and its association with MIGS version 2.0 compliance [10].

**Table 2:** Project information

MIGS ID	Property	Term
MIGS-31	Finishing quality	High-quality draft
MIGS-28	Libraries used	One pair-end 500 bp library and one pair-end 2 Kb library
MIGS-29	Sequencing platforms	Illumina HiSeq 2000
MIGS-31.2	Fold coverage	130 × (based on 500 bp library), 65 × (based on 2 Kb library)
MIGS-30	Assemblers	SOAP *denovo*
MIGS-32	Gene calling method	RAST
	Locus Tag	LT39
	Genbank ID	JROF00000000
	Genbank Date of Release	November 17, 2014
	GOLD ID	Gi0079167
	NCBI Project ID	261874
	BIOPROJECT	PRJNA261874
MIGS 13	Source Material Identifier	JCM 18547
	Project relevance	Phyloenetic diversity, Study of the archaeal diversity in a salt mine

### Growth conditions and genomic DNA preparation

*H. salifodinae* KCY07-B2^T^ was cultivated aerobically on 37 °C for 4 days in NOM-3 medium, which contains (per liter distilled water) 5.4 g KCl, 0.3 g K_2_HPO_4_, 0.25 g CaCl_2_, 0.25 g NH_4_Cl, 26.8 g MgSO_4_ · 7H_2_O, 23.0 g MgCl_2_ · 6H_2_O, 184.0 g NaCl, 1.0 g yeast extract, 0.25 g fish peptone, 0.25 g sodium formate, 0.25 g sodium acetate, 0.25 g sodium lactate and 0.25 g sodium pyruvate (adjusted to pH 7.0 with 1 M NaOH) [3]. Genomic DNA was extracted using the method described by Marmur [11]. The purity, quality and the concentration of genomic DNA preparation were analyzed by 0.7 % agarose gel electrophoresis with λ-*Hin*d III digest DNA Marker (TaKaRa, Dalian, China) and measured using a NanoDrop 1000 Spectrophotometer (Thermo Fisher Scientific Inc., USA).

### Genome sequencing and assembly

The genome of *H. salifodinae* KCY07-B2^T^ was sequenced using Solexa paired-end sequencing technology (HiSeq2000 system, Illumina, Inc., USA) [12]. A shotgun library was constructed with a 500 bp-span paired-end library (~500 Mb available reads, ~130-fold genome coverage) and a 2000 bp-span paired-end library (~250 Mb available reads, ~65-fold genome coverage). The sequence data from an Illumina HiSeq 2000 were assembled with SOAP*denovo* v.1.05 [13–15]. The final assembly identified 83 contigs and 81 scaffolds (the minimum length is 523 bp) generating a genome size of 4.35 Mb. The quality of the sequencing reads data was estimated by G + C content and sequencing depth correlation analysis.

### Genome annotation

The tRNAs and rRNAs were identified using tRNAscan-SE [16], RNAmmer [17] and Rfam database [18]; The open reading frames and the functional annotation of translated ORFs were predicted and achieved by using the RAST server online [19, 20]. Classification of some predicted genes and pathways were analyzed using COGs [21, 22] and KEGG [23–25] databases. Meanwhile, we used CRISPRs web server [26] to predict CRISPRs and InterPro [27, 28] to obtain the GO annotation with the database of Pfam [29].

To estimate the mean level of nucleotide sequence similarity at the genome level between *e* KCY07-B2^T^ and the genus *Halopier* genomes available to date (*H. xanaduensis* SH-6^T^, *H. djelfamassiliensis* IIH2^T^ and *H. goleamassiliensis* IIH3^T^), we compared the ORFs only using comparison sequence based in the server RAST [19] at a query coverage of ≥60 % and a minimum nucleotide length of 100 bp.

## Genome properties

The draft genome sequence of *H. salifodinae* KCY07-B2^T^ revealed a genome size of 4,350,718 bp (scaffold length) with a 65.41 % G + C content. Of the 4254 predicted genes, 4204 were protein-coding genes, and 50 were rRNA genes. There were one 16S rRNA gene, two 23S rRNA genes and two 5S rRNA genes. A total of 2887 genes (68.67 %) were assigned a putative function (Table 3). Table 4 showed the distribution of genes into COG functional categories.

**Table 3:** Genome statistics of *Halopiger salifodinae* KCY07-B2^T^, including nucleotide content and gene count levels

Attribute	Value	% of total^a^
Genome size (bp)	4,350,718	100.00
DNA coding (bp)	3,567,421	82.00
DNA G + C (bp)	2,845,805	65.41
DNA scaffolds	81	
Total genes	4254	100.00
Protein coding genes	4204	98.82
RNA genes	50	1.18
Pseudo genes	not determined	not determined
Genes in internal clusters	not determined	not determined
Genes with function prediction	2561	60.20
Genes assigned to COGs	2887	67.87
Genes assigned Pfam domains	2694	63.33
Genes with signal peptides	122	2.9
Genes with transmembrane helices	910	21.39
CRISPR repeats	3	0.07

^a^The total is based on either the size of the genome in base pairs or the total number of protein coding genes in the annotated genome

**Table 4:** Number of genes associated with the 25 general COG functional categories

Code	value	% age^a^	Description
J	175	4.16	Translation, ribosomal structure and biogenesis
A	1	0.02	RNA processing and modification
K	175	4.16	Transcription
L	120	2.85	Replication, recombination and repair
B	6	0.14	Chromatin structure and dynamics
D	25	0.59	Cell cycle control, Cell division, chromosome partitioning
V	35	0.83	Defense mechanisms
T	131	3.12	Signal transduction mechanisms
M	118	2.81	Cell wall/membrane biogenesis
N	16	0.38	Cell motility
U	19	0.45	Intracellular trafficking and secretion
O	131	3.12	Posttranslational modification, protein turnover, chaperones
C	255	6.07	Energy production and conversion
G	200	4.76	Carbohydrate transport and metabolism
E	306	7.28	Amino acid transport and metabolism
F	77	1.83	Nucleotide transport and metabolism
H	159	3.78	Coenzyme transport and metabolism
I	115	2.74	Lipid transport and metabolism
P	215	5.11	Inorganic ion transport and metabolism
Q	60	1.43	Secondary metabolites biosynthesis, transport and catabolism
R	550	13.08	General function prediction only
S	299	5.45	Function unknown
-	1317	31.33	Not in COGs

^a^The total is based on the total number of protein coding genes in the genome

## Insights from the genome sequence

Strain *H. salifodinae* KCY07-B2^T^ was isolated from a salt mine sample. The experiments showed this strain could grow at 2.9–3.4 M NaCl for optimal growth, and the cells lysed in distilled water. So the analysis of the genome sequence focused on the adaption mechanism of the halophilic archaea in hypersaline-environments. Strain *H. salifodinae* KCY07-B2^T^ mainly utilized “the salt-in strategy” to maintain osmotic balance. According to the annotation of genome sequence, Trk system potassium uptake protein were found, which were responsible for K^+^ uptake and transport, including 9 copies *TrkH* genes and 5 copies *TrkA* genes. Five copies of Kef-type K^+^ transport proteins, one copy glutathione-regulated potassium-efflux protein KefB and 8 pH adaptation potassium efflux system proteins were found that were related to K^+^ efflux. And there also existed 8 copies of potassium channel proteins. In addition, the genome contains 13 copies of Na^+^/ H^+^ antiporter proteins related to Na^+^ efflux. The genome of strain *H. salifodinae* KCY07-B2^T^ contains 12 genes related to the synthesis and transport of the compatible-solute glycine betaine for resistance to osmotic stress including: 7 choline-sulfatases, 2 high-affinity choline uptake protein BetTs, 2 glucose-methanol-choline oxidoreductase and 1 glycine betaine transporter OpuD coding genes. These proteins were also related to the metabolic pathway converting choline sulfate to glycine betaine. All these proteins and systems mentioned played an important role in the adaption of osmotic stress in high salt environment.

Currently, three genomes from *Halopiger* species are available. Here, we compare the genome of strain *H. salifodinae* KCY07-B2^T^ with strains *H. xanaduensis* SH-6^T^, *H. djelfamassiliensis* IIH2^T^ and *H. goleamassiliensis* IIH3^T^ (Table 5). The size of genome of *H. salifodinae* KCY07-B2^T^ (4.35 Mb) is similar to *H. xanaduensis* SH-6^T^ (4.35 Mb) but larger than that of *H. djelfamassiliensis* IIH2^T^ (3.77 Mb) and *H. goleamassiliensis* IIH3^T^ (3.90 Mb). The G + C content of *H. salifodinae* KCY07-B2^T^ (65.41 %) is similar to *H. xanaduensis* SH-6^T^*(*65.18 %) and higher than that of *H. djelfamassiliensis* IIH2^T^ (64.30 %) but lower than that of *H. goleamassiliensis* IIH3^T^ (66.06 %). In addition, *H. salifodinae* KCY07-B2^T^ shares a mean genomic sequence similarity of 79.74 %, 80.16 % and 79.17 % with strains *H. xanaduensis* SH-6^T^, *H. djelfamassiliensis* IIH2^T^ and *H. goleamassiliensis* IIH3^T^, respectively.

**Table 5:** Genomic comparison of *H. salifodinae* KCY07-B2T with three other *Halopiger* species^a^

Species	Strain	Genome accession number	Genome size (Mb)	G + C content
*H. salifodinae*	KCY07-B2^T^	JROF00000000	4.35	65.41
*H. xanaduensis*,	SH-6^T^	NC_015666	4.35	65.18
*H. djelfamassiliensis*	IIH2^T^	PRJEB1777	3.77	64.30
*H. goleamassiliensis*	IIH3^T^	PRJEB1780	3.90	66.06

^a^Species and strain names, genome accession numbers, sizes and G + C contents

## Conclusions

Strain KCY07-B2^T^ is the third member of the genus *Halopiger* to be described and the fourth whose genome sequence report is available. These data will provide a new perspective of how microorganisms adapt to halophilic environments, and may also provide a pool of functional enzymes that work at higher salty.

## Abbreviations

NCBI: National Center for Biotechnology Information
EMBL: European Molecular Biology Laboratory
DDBJ: DNA Data Bank of Japan
BLASTN: Basic Local Alignment Search Tool for Nucleotide
MIGS: Minimum Information about a Genome Sequence
RAST: Rapid Annotations using Subsystems Technology
COG: Cluster of Orthologous Groups of proteins
KEGG: Kyoto Encyclopedia of Genes and Genomes
CRISPR: Clustered Regularly Interspaced Short Palindromic repeat sequences
GO: Gene Ontology
DNA: Deoxyribonucleic Acid
16S rRNA: ribosomal Ribonucleic Acid
JCM: Japan Collection of Microorganisms
CGMCC: China General Microbiological Culture Collection Center
*H. salifodinae* KCY07-B2^T^: *Halopiger salifodinae* KCY07-B2^T^
*H. xanaduensis* SH-6^T^: *Halopiger xanaduensis* SH-6^T^
*H. aswanensis* 56^T^: *Halopiger aswanensis* 56^T^
*H. djelfamassiliensis* IIH2^T^: *Halopiger djelfamassiliensis* IIH2^T^
*H. goleamassiliensis* IIH3^T^: *Halopiger goleamassiliensis* IIH3^T^
